# Dengue infection modulates locomotion and host seeking in *Aedes aegypti*

**DOI:** 10.1371/journal.pntd.0008531

**Published:** 2020-09-10

**Authors:** Anaïs K. Tallon, Marcelo G. Lorenzo, Luciano A. Moreira, Luis E. Martinez Villegas, Sharon Rose Hill, Rickard Ignell

**Affiliations:** 1 Disease Vector Group, Department of Plant Protection Biology, Swedish University of Agricultural Sciences, Alnarp, Sweden; 2 Vector Behaviour and Pathogen Interaction Group, Instituto René Rachou, Fundação Oswaldo Cruz, Belo Horizonte, Minas Gerais, Brazil; 3 Mosquitos Vetores: Endossimbiontes e Interação Patógeno-Vetor, Instituto René Rachou-Fiocruz, Belo Horizonte, Minas Gerais, Brazil; Australian Red Cross Lifelood, AUSTRALIA

## Abstract

Pathogens may manipulate their human and mosquito hosts to enhance disease transmission. Dengue, caused by four viral serotypes, is the fastest-growing transmissible disease globally resulting in 50–100 million infections annually. Transmission of the disease relies on the interaction between humans and the vector *Aedes aegypti* and is largely dependent on the odor-mediated host seeking of female mosquitoes. In this study, we use activity monitors to demonstrate that dengue virus-1 affects the locomotion and odor-mediated behavior of *Ae*. *aegypti*, reflecting the progression of infection within the mosquito. Mosquitoes 4–6 days post-infection increase locomotion, but do not alter their odor-driven host-seeking response. In contrast, females 14–16 days post-infection are less active, yet more sensitive to human odors as assessed by behavioral and electrophysiological assays. Such an increase in physiological and behavioral sensitivity is reflected by the antennal-specific increase in abundance of neural signaling transcripts in 14 days post-infection females, as determined by transcriptome analysis. This suggests that the sensitivity of the mosquito peripheral olfactory system is altered by the dengue virus by enhancing the overall neural responsiveness of the antenna, rather than the selective regulation of chemosensory-related genes. Our study reveals that dengue virus-1 enhances vector-related behaviors in the early stages post-infection that aid in avoiding predation and increasing spatial exploration. On the other hand, at the later stages of infection, the virus enhances the host-seeking capacity of the vector, thereby increasing the risk of virus transmission. A potential mechanism is discussed.

## Author summary

The mosquito *Aedes aegypti* is a major urban vector for human pathogens, including dengue, the most rapidly-spreading infectious disease globally. We show that one of the four strains of the dengue virus differentially, and inversely, affects the locomotion and odour-mediated behaviour of female *Aedes aegypti*. During the early stages after infection, female mosquitoes show an increased locomotion activity suggesting that the virus likely promotes mosquito dispersion and increases its spatial exploration. In contrast, in the later infective stages, dengue infection modulates the mosquito’s host-seeking capacity, by enhancing the physiological and behavioural sensitivity to human odors, thus increasing the risk of virus transmission. At this stage of infection, neural signalling gene expression is enhanced, providing a mechanism for the increased sensitivity to human odor, while genes involved in chemosensation and neuromodulation are likely playing a secondary role. A better understanding of alterations of mosquito host-seeking behavior upon pathogen infection will contribute to the development of novel control strategies against vector borne diseases.

## Introduction

Pathogens and parasites have the capacity to manipulate their hosts, enhancing the cycle of disease transmission [1–3]. Disease agents are able to impact the physiology, behavior, reproduction and survival of their insect host [4–7]. Similar effects have been shown in the primary hosts, in which infection results in changes in attraction and defensive behavior, as well as in the vascular and immune systems [8–11]. Such manipulation is increasingly being described for the interaction between blood feeding insects and their vertebrate hosts. This has significant implications for the epidemiology and control of vector-borne diseases [12,13].

Behaviors, such as locomotion, flight, host seeking, probing and feeding, are directly linked to the capacity of blood feeding insects to transmit disease [14,15]. Pathogen or parasite infection may result in a change in flight and locomotion [16,17], induced through either systemic or direct manipulation of the neural circuits underlying these behaviors. Infected insects are also more avid in locating a blood meal, achieved through changes in attraction and feeding pattern on suitable hosts [18–20], or in a change in host preference [21]. The change in feeding pattern is reflected by an increased probing time and/or frequency and persistence of blood feeding, resulting in an enhanced transmission through an increased contact between vector and host [19,22–26]. The behavioral modulation by disease agents does not appear to be static, as demonstrated for *Plasmodium*-infected malaria mosquitoes [20,22,27]. Recent studies indicate that pathogens and parasites may target the many conserved pathways underlying particular behavioral traits to enhance transmission [28–30], suggesting that the olfactory system is affected through the change in chemosensory gene expression.

The behavior of the primary urban vector of dengue, *Aedes aegypti*, is manipulated by at least two of the four virus serotypes, although at different time scales [6,8,24,31]. Dengue virus-2 (DENV-2) infected mosquitoes display an increased locomotion behavior, 8–10 days post-infection (dpi) [17], and a change in feeding pattern 14 dpi [32), which appear to be regulated through a change in transcript abundance of select chemosensory genes [33]. In contrast, the effect of DENV-3 infection on probing time and persistence occurs already at 8 dpi [24], suggesting that the dengue virus affects different pathways during the course of infection. While DENV-2 infection has been shown to affect the odor-mediated oviposition behavior of gravid *Ae*. *aegypti* [34], such an effect has not yet been described for the characteristic odor-mediated behavior of host seeking females.

In this study, we aimed to assess how dengue infection affects vector-related behaviors by demonstrating that DENV-1 differentially, and inversely, affects the locomotion and odor-mediated behavior of its mosquito host, an effect dependent on the extrinsic incubation period of the virus. The observed increase in locomotor activity of infected *Ae*. *aegypti* is described at the first week post-infection, whereas an increased host responsiveness is shown at the second week post-infection. While phenotypic effects of pathogen infection are increasingly being described at the behavioral level, little is known about the physiological and molecular mechanisms underlying this modulation. A transcriptome analysis emphasizes that genes involved in neural signaling pathways likely underpin the observed behavioral and physiological phenotypic effects, with genes involved in chemosensation and neuromodulation likely playing a secondary role.

## Methods

### Mosquito rearing

*Aedes aegypti* (BR strain), a colony replenished periodically with eggs collected from the Urca neighborhood, Rio de Janeiro, Brazil, were reared at 25 ± 1°C, 70 ± 10% relative humidity, and at a photoperiod of 12 h light: 12 h dark. Larvae were fed with fish food pellets (Tetra GmbH, Melle, Germany), and pupae were transferred into mesh cages (30 cm × 30 cm × 45 cm) until adult emergence. Adult mosquitoes were given *ad libitum* access to a 10% sucrose solution. Females used for these experiments correspond with the F2 generation of the collected wild type mosquitoes. At five days post-emergence, sexes were separated, and females provided a human blood meal. Females were fed on human blood, as the sole source of blood, to maximise the fitness of the colony [35] and the DENV-1 virus [36]. Mosquitoes were sugar-starved 20 h before both tissue collection and behavioral assays.

### Dengue virus preparation and feeding

Dengue virus serotype 1 (DENV-1, strain Fiocruz, isolated during the outbreak of 2015, in Contagem, Minas Gerais, Brazil) stocks were cultured in a mock medium, and maintained through cell lines (C6/36 *Aedes albopictus*) that were grown in Leibowitz L-15 medium supplemented with 10% fetal bovine serum (Gibco) at 28°C, and then frozen at -80°C, as previously described [37]. Prior to feeding, mosquitoes were starved for 20 h, and 1 ml aliquots of the virus stock (≥2.5 × 10^6^ CFU ml^-1^) were thawed and diluted into 500 μl of human blood [38]. The blood-virus mixture was transferred to a membrane feeder, which was covered with porcine intestine, and maintained at 37°C. Female mosquitoes were orally infected by feeding on the DENV-1-infected blood for 1 h. As a control, female mosquitoes from the same cohort were fed on human blood, containing only the mock medium. After blood feeding, mosquitoes were anesthetized by CO_2_, and only fully engorged individuals were kept for further experiments.

### Dengue viral load analysis

The success of the experimental infections was individually tested on a total of 502 mosquitoes used for subsequent transcriptome analysis and electrophysiological experiments. The success of infection of mosquitoes used in behavioral assays could not be tested due to ethical regulation concerning the handling of infected insects. Total viral RNA was extracted from both infected and non-infected individual mosquitoes (High Pure Viral Nucleic Acid Kit, Roche, Germany). To this end, the body tissues were disrupted with beads and homogenized using a high-throughput cell disrupter (Mini-Beadbeater-96, BioSpec Products Inc. US). Both RNA extraction and DNase digestion steps were performed using High Pure Viral Nucleic Acid kit (Roche, Mannheim, Germany), following the manufacturer’s protocol, and the total amount of RNA was quantified with a NanoDrop 2000c (ThermoFisher Scientific, USA). The qPCR assay was performed using previously published probes and primers for DENV-1 and the *Ae*. *aegypti* house-keeping gene *RpS17* [39]. The viral load was determined by absolute quantification based on DENV-1 standard curves and detection thresholds, as previously described [38]. For each sample, two technical replicates were performed, and “no template” controls were run alongside the samples. The total reaction volume was 10 μl, consisting of 5X RT-qPCR Reaction Mix (LightCycler Multiplex RNA Virus Master, Roche; Mannheim, Germany), 0.5 μM of each forward and reverse primer, 0.2 μM for each probe, 200X RT enzyme solution and approximately 50 ng of RNA sample. Reactions were performed in a LightCycler 96 Instrument (Roche, Mannheim, Germany) with the following conditions: 50°C for 10 min, 95°C for 30 s, followed by 45 cycles of 95°C for 5 s and 60°C for 30 s. For each successfully infected mosquito, the average quantitation cycle (Cq) value was calculated between the two technical replicates, and the median Cq of all the samples was determined (Cq = 24.455). RNA samples presenting a Cq higher than the median (Cq > 24.455) were considered as having a low level of infection, while samples with a lower Cq (Cq < 24.455) were considered as having a high level of infection (S4 Data). While this represents an arbitrary division of infection levels, the high level of infection encompasses the range found naturally [40].

### Mosquito locomotor activity

To verify and further extend the demonstrated increase in behavioral activity of dengue infection in *Ae*. *aegypti* [17], 4 days post-infection (dpi) and 14 dpi females were placed in DAM2 *Drosophila* activity monitors (TriKinetics Inc. Waltham, MA, USA), along with non-infected females of the same age, and the locomotor activity was recorded for 3 d. The first 12 h of recording was considered as the necessary time for the mosquitoes to adapt to the experimental environment and was not presented in the results. Females were individually positioned in glass tubes (65 mm × 7 mm) using an aspirator (Clarke, St Charles, IL, USA). Cotton plugs, saturated with 10% sucrose solution, were placed at one end of the glass tubes in order to provide mosquitoes with a food source, while the other end was sealed with mesh caps. The glass tubes were then positioned in the activity monitors, kept in the biosafety lab used for infected mosquitoes, at 25 ± 1°C, 70 ± 10% relative humidity, and at a photoperiod of 12 h light: 12 h dark. Following an acclimation period of 30 min, the number of times each individual mosquito interrupted the beam was recorded in intervals of 30 min for three consecutive days, using the DAMSystem3 Software (TriKinetics Inc. Waltham, MA, USA). A total of 1536 mosquitoes were tested.

Statistical analysis was made on the total and per time point locomotor activity (the latter being a per individual number of beam breaks accumulated every 30 min). A redundancy analysis (RDA) and permutational multivariate analysis of variance (PERMANOVA) were used to describe and measure the variation in locomotion profiles of all individual infected and non-infected mosquitoes at each time point, as described previously [41]. In brief, the RDA allows for pairwise comparisons among individuals at each time point, throughout the experiment, and for identifying the existance and quantifying the similarities or disimilarities in activity profiles, in the context of pre-determined explanatory variables, *i*.*e*., infection and total activity. Any similarities or disimilarities are then assessed by PERMANOVA, which not only returns the statistical significance of the proposed variable, but also the effect size (R^2^) [41]. The first day of recording was removed from the analysis, as per common practice. The data of individuals that died during the experiments were removed from the analysis.

To assess the average overall locomotor activity of all the mosquitoes across the two and a half days of recording, the William’s mean was used [42]. This geometric mean (log (n+1)) was calculated for all the recordings every 30 min, and minimizes the influence of low/high values and inflated numbers of zeros on the data distribution. To compare the William’s means, a repeated measures two-way ANOVA with a Tukey’s *post hoc* analysis was used to compare the overall locomotion activity every 30 min at 4.5–6 dpi and 14.5–16 dpi.

### Odor-mediated locomotion behavior

To assess whether dengue infection affected the odor-mediated locomotion of *Ae*. *aegypti*, DAM2 *Drosophila* activity monitors, equipped with MAN2 gas distribution manifolds (TriKinetics), were used. A synthetic human odor blend, identified from the body volatiles of human volunteers [43] was used to stimulate dengue-infected and non-infected *Ae*. *aegypti* female mosquitoes. This blend consists of butyl acetate, 1-hexanol, 3-octanol, *R*-1-octen-3-ol, 2-nonanol, decanal, octanal, nonanal, phenol, benzaldehyde, acetophenone, limonene, sulcatone, linalool, and α-terpineol, (4.8 : 5.7 : 18 : 1 : 1.1 : 115 : 7.7 : 76 : 1 : 4.4 : 0.4 : 150 : 51 : 5.7 : 3.3) [37] Either synthetic human odor (44 ng h^-1^) or pentane, used as a control, was released from wick dispensers [44] placed inside wash bottles and then delivered to the manifolds through Teflon tubing at 2.2 l min^-1^, using a stimulus controller (CS-55; Syntech, Kirchgarten, Germany). Treatment and control assays were run in parallel for each age cohort. The activity of 6 dpi and 14 dpi mosquitoes, as well as that of non-infected counterparts, handled as above, was recorded every 30 s, between Zeitgeber Time (ZT) 7–9 and ZT 12–14, identified as the peak host-seeking activity period of *Ae*. *aegypti* [17,45]. A total of 1 536 mosquitoes was tested. The total locomotor activity and corresponding individual profiles were calculated and compared among treatments and infection status through an RDA and permutational MANOVA test. The data of individuals that died during the experiments were removed from the analysis.

### Electroantennographic analysis

Female mosquitoes were starved (provided with water only) 24 h prior to electrophysiological analysis. Individual mosquitoes were anaesthetized on ice, after which an antennal preparation was made using the excised head. After cutting the distal segment of one of the antennae, the preparation was mounted between two gel electrodes. Both electrodes were covered with electrically conductive gel (Spectra 360, Parker Laboratories, USA), and connected to a high impedance DC amplifier interface box (IDAC-232, Syntech) via a pre-amplifier probe (Syntech). A continuous charcoal-filtered and humidified airflow (2.2 l min^-1^) was passed through a glass tube and flushed over the antennal preparation, positioned 10 mm from the tube outlet. Odor stimuli were produced by loading filter papers (1 cm × 2 cm), inserted into glass Pasteur pipettes, with 10 μl of either the synthetic human odor, diluted in pentane by decadic steps, or pentane as a control. Pipettes with formulated filter papers were kept for 15 min in a fume hood to allow for solvent evaporation. Using a stimulus controller (CS-55, Syntech), a 0.5 s (1.7 l min^-1^) air-puff was passed through the stimulus pipette and embedded into the continuous airstream through a hole in the glass tube at 10 cm distance from the preparation, maintaining a constant airflow through pulse flow compensation. Stimuli were delivered from low to high concentration sequentially, with 30 s in-between stimulus intervals, with pentane used as a control between each odor stimulation. Individual antennae were used only once for each series of odor stimulations, which accounted for a single replicate. Between 15 and 20 replicates were performed for each infection state and age. All recordings were performed during the peak activity period.

The amplitude of the antennal response to human odor was compared at 6 dpi and 14 dpi between infected and non-infected individuals using repeated measures two-way ANOVA, considering the two hypothesized explanatory variables “concentration” and “infection status”. For each individual, the relative amplitude of the antennal response to an odor stimulus was normalized by subtracting the average response to pentane presented before and after the odor stimulus, from the response.

### Antennal transcriptome analysis

Antennae of both 14 dpi and similarly aged non-infected adult females were collected during three hours within the activity peak. Individuals were cold-anaesthetized, the antennae removed using forceps, and then immediately transferred into 24-well cell culture plates (Sarstedt, Germany) containing 500 μl RNAlater (Thermo Fisher Scientific, Sweden). Both antennae of the same individual were placed in the same well. All samples were stored at room temperature for 24 h, and then transferred to -80°C until RNA extraction. Simultaneously, whilst dissecting the antennae, the bodies of infected females were placed in separate wells in 24-well plates and subsequently subjected to qPCR analysis to quantify viral load, as described above. RNA extraction and DNase I digestion were performed using the RNeasy Mini Kit (Qiagen, Sweden) following the manufacturer’s protocol, on the antennae of successfully infected females. Aliquots of RNA were tested for both quantity and quality on an Agilent 2100 Bioanalyzer (Agilent Technologies, Waldbronn, Germany). Following the quantification of viral load (above), antennal RNA samples of successfully infected individuals were pooled in tubes containing a total of 50 antennae, according to the level of infection, and stored at -80°C.

### Sequencing, read mapping and gene annotations

A total of 19 samples (9 non-infected, 5 with low levels of infection, and 5 with high levels of infection) were shipped on dry ice to the Max Planck-Genome-Center (Cologne, Germany, https://mpgc.mpipz.mpg.de/home/) for complementary deoxyribonucleic acid (cDNA) library construction and RNA-Seq quantification (Illumina HiSeq 2000). Library construction was performed using the Ovation SoLo RNA-Seq System Core Kit (NuGEN, Leek, The Netherlands), following the depletion of rRNA using Solo AnyDeplete Primer Mix–Custom for *Ae*. *aegypti* (NuGEN). Prior to the quantitative analysis, quality controls were performed according to Max Planck-Genome-Center standards. Quantitative paired-end sequencing of antennal RNA from each of the 19 libraries generated on average over 17 million cleaned reads, which were mapped to the reference genome (AaegL5) obtained from NCBI using CLC Genomics Workbench version 11.0.2 (Qiagen, Århus, Denmark).

### RNA-Seq and differential expression analyses

As previously described [46,47], a quantile-normalization was performed on each library in order to permit the detection of transcripts with low expression levels, and a threshold of >1 transcript per kilo base million (TPM), commonly used to reduce noise [46,47], was applied to remove genes considered as unreliably detected from subsequent analysis. Similar to DESeq, CLC allowed the estimation of transcript abundance and tested differential expression at the gene level using a Generalized Linear Model (GLM). From the whole transcriptome dataset, transcript abundance in units of TPM was compared between non-infected, low infection, and high infection levels, and candidate genes-of-interest were identified according to their differential fold change (FC > 2) in transcript abundance, using the beta-binomial Baggerley’s test [48] and a false discovery rate (FDR)-corrected p-value (*P*) of < 0.05 [49].

## Results

### Dengue infection differentially affects locomotor activity

**Fig 1:**
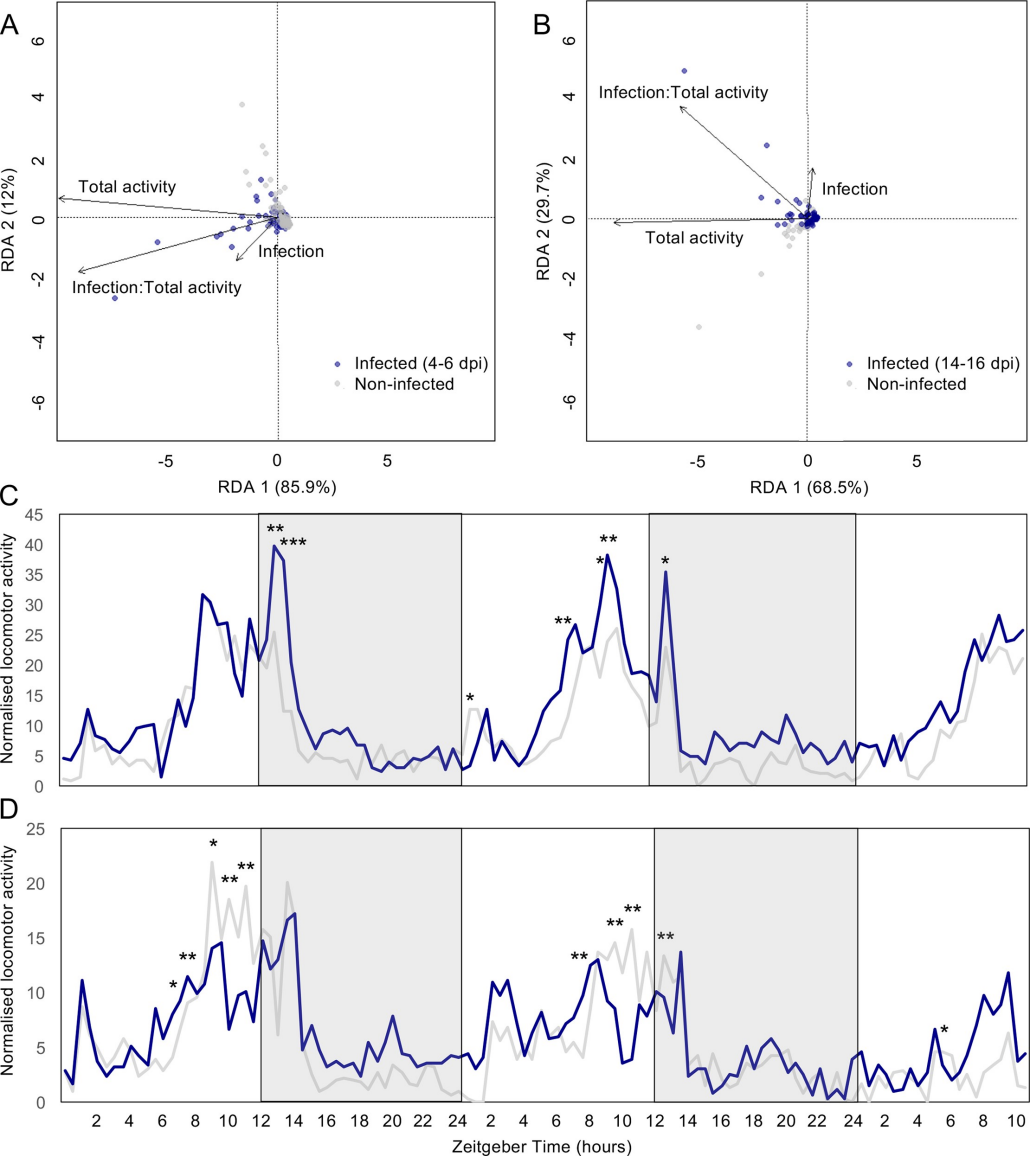
Dengue infection differentially affects locomotor activity. Individual locomotor profiles (n = 317) were assessed for variation using a redundancy analysis, revealing an effect of DENV-1 in 4.5–6 days post-infection (dpi) A) but not 14.5–16 dpi; B) female *Ae*. *aegypti*. Each dot represents the observed activity profile for each individual mosquito throughout the experiment. The position within the ordination is determined by the pairwise Euclidean distance among all the objects in the dataset. Arrows indicate the vectors describing the variance with regards to each binary/quantitative explanatory variable (infection, total activity). **C & D**) Average diel locomotor activity profiles of non-infected (grey) and either 4.5–6 days post-infection (dpi) (blue; C) or 14.5–16 dpi (blue; D) female *Ae*. *aegypti*, calculated using the William’s means. The grey boxes represent the 12 h scotophase (C, D). Significances were determined using a repeated measures two-way ANOVA followed by a pairwise comparison via Tukey’s *post hoc* test. Asterisks indicate level of significance (* P < 0.05; ** P < 0.01; *** P < 0.001).

Infection with DENV-1 differentially affected the locomotor activity of 4.5–6 dpi and 14.5–16 dpi *Ae*. *aegypti* (Fig 1), when compared with non-infected insects. A redundancy analysis (RDA) showed that at 4.5–6 dpi, a total of 15.7% of the variation, which was observed between the individual locomotion profiles, was constrained by the two hypothesized explanatory variables (infection status and total activity). From this constrained variance, 97.9% was represented on the two axes of the triplot (Fig 1A). The angle between the vectors “total activity” and “infection status” was less than 90° revealing a positive correlation between the two variables (Fig 1A), and suggesting that dengue-infected females (4.5–6 dpi) move differentially compared with their non-infected counterparts. This was confirmed following a permutational MANOVA analysis, which revealed that the proportion of observed variance between infected and non-infected mosquitoes was significantly explained by infection status (R^2^ = 0.8, df = 1, P = 0.014). While similar patterns of locomotion activity were observed over a two-day period, a two-way ANOVA confirmed a significant difference in the overall activity between infected and non-infected females (F = 37.081, df = 1, P < 0.001; Fig 1C). Dengue infection induced an increase in locomotion activity throughout the time of recording, particularly during dawn and dusk with the peak activity occurring in the last hours of the photophase (ZT 8–10) (Fig 1C). The infection status of these individuals was extrapolated to be ca. 80%, based on that determined for the electrophysiology and gene expression analyses (average = 80.9%).

At 14.5–16 dpi, 14.2% of the variation observed between individual locomotion profiles was constrained in the analysis, out of which 98.2% was represented on the two axes of the RDA (Fig 1B). However, infection status did not significantly influence (PERMANOVA: R^2^ = 0.05, df = 1, P > 0.05) the variance measured when comparing between the overall locomotion profile of 14.5–16 dpi individuals and age-matched non-infected females. A two-way ANOVA revealed that while infection had no effect on the overall diel locomotion activity (F = 2.501, df = 1, P > 0.05), there was a reduction in the peak of locomotion occurring before the transition light on/off (ZT 8–10), with non-infected mosquitoes being significantly more active than 14.5–16 dpi mosquitoes (Fig 1D).

### Dengue infection affects response to host odor

**Fig 2:**
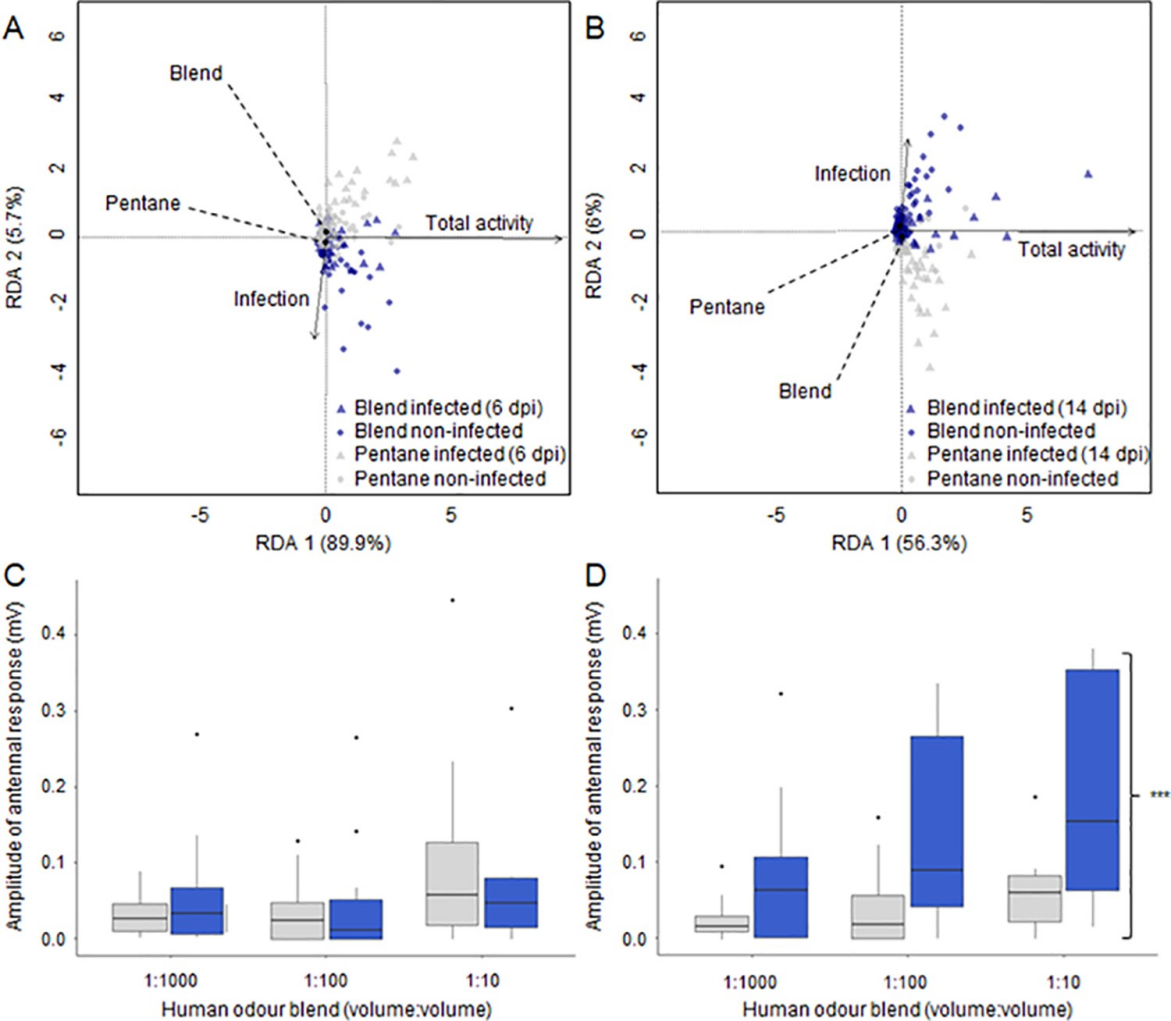
Dengue infection affects the behavioral and physiological response to human odor. Individual odor-mediated locomotor profiles (n = 983) were assessed for variation using a redundancy analysis, revealing no effect of DENV-1 in 6 days post-infection (dpi) female *Ae*. *aegypti* **A**), while the virus elicited altered locomotor profiles in the presence of the synthetic human odor (blend) in 14 dpi females **B**). Each dot represents the observed activity profile for each individual mosquito throughout the experiment. The position within the ordination is determined by the pairwise Euclidean distance among all the objects in the dataset. Arrows indicate the vectors describing the variance with regards to each quantitative explanatory variable (infection, total activity), while the centroids represent the qualitative variables (blend, pentane control). The electrophysiological dose-response profiles (n = 56) of the antennae of 6 dpi (**C**) and 14 dpi (**D**) female *Ae*. *aegypti* to the synthetic human odor blend and the pentane control. The whiskers in the boxplots represent the 95% confidence intervals. The significant difference in sensitivity in the antennae of 14 dpi mosquitoes, based on a two-way ANOVA, is indicated to the right (P < 0.001).

The average odor-mediated locomotor activity was not significantly different between infected and non-infected female mosquitoes, in response to either the synthetic human odor blend or the pentane control (6 dpi: n_human odour_ = 127, n_pentane_ = 128, t = -0.52, df = 467, P > 0.05, and 14 dpi: n_human odour_ = 128, n_pentane_ = 128, t = 0.12, df = 499, P > 0.05 respectively). However, at 14 dpi, an RDA revealed that both infection status and total activity were relevant and correlated explanatory variables to describe the variation in individual locomotion profiles, unlike at 6 dpi (Fig 2A and 2B). A permutational MANOVA analysis revealed that 89.2% of the variance in activity profiles observed between non-infected and 14 dpi individuals is explained by the hypothesized explanatory variables, while only 12.3% of the variance is explained at 6 dpi. Interestingly, at 14 dpi, the interaction between infection status and the nature of the stimulus significantly explained the variation observed in locomotion profiles of individuals (F = 1.27, df = 2, P = 0.011).

### Dengue infection alters antennal sensitivity

Both infected and non-infected *Ae*. *aegypti* displayed a dose-dependent response to the synthetic human odor blend, irrespective of age (6 dpi: F = 5.46, df = 2, P = 0.0062; 14 dpi: F = 8.38, df = 2, P = 0.0007) (Fig 2C and 2D). While DENV-1 infection had no significant effect on the antennal response of infected mosquitoes 6 dpi (P > 0.05) infection significantly increased the antennal response to the synthetic human odor in mosquitoes 14 dpi, when compared to similar-aged controls (F = 7.81, df = 1, P = 0.009) (Fig 2C and 2D).

### Dengue infection induces differential transcript abundances

Quantitative paired-end sequencing of antennal RNA from each of the libraries generated an average mapping of over 17 million cleaned reads per library (S1 Table). A total of 19,241 transcripts were detected in the antennae of both 14 dpi and non-infected females of the same age, out of which 16,550 transcripts were reliably detected above the threshold of 1 transcript per million mapped reads (TPM) (S1 Table) demonstrating a good level of coverage. In addition, the 100 least abundant genes reliably detected in previous studies [46,47], were also found in this study.

**Fig 3:**
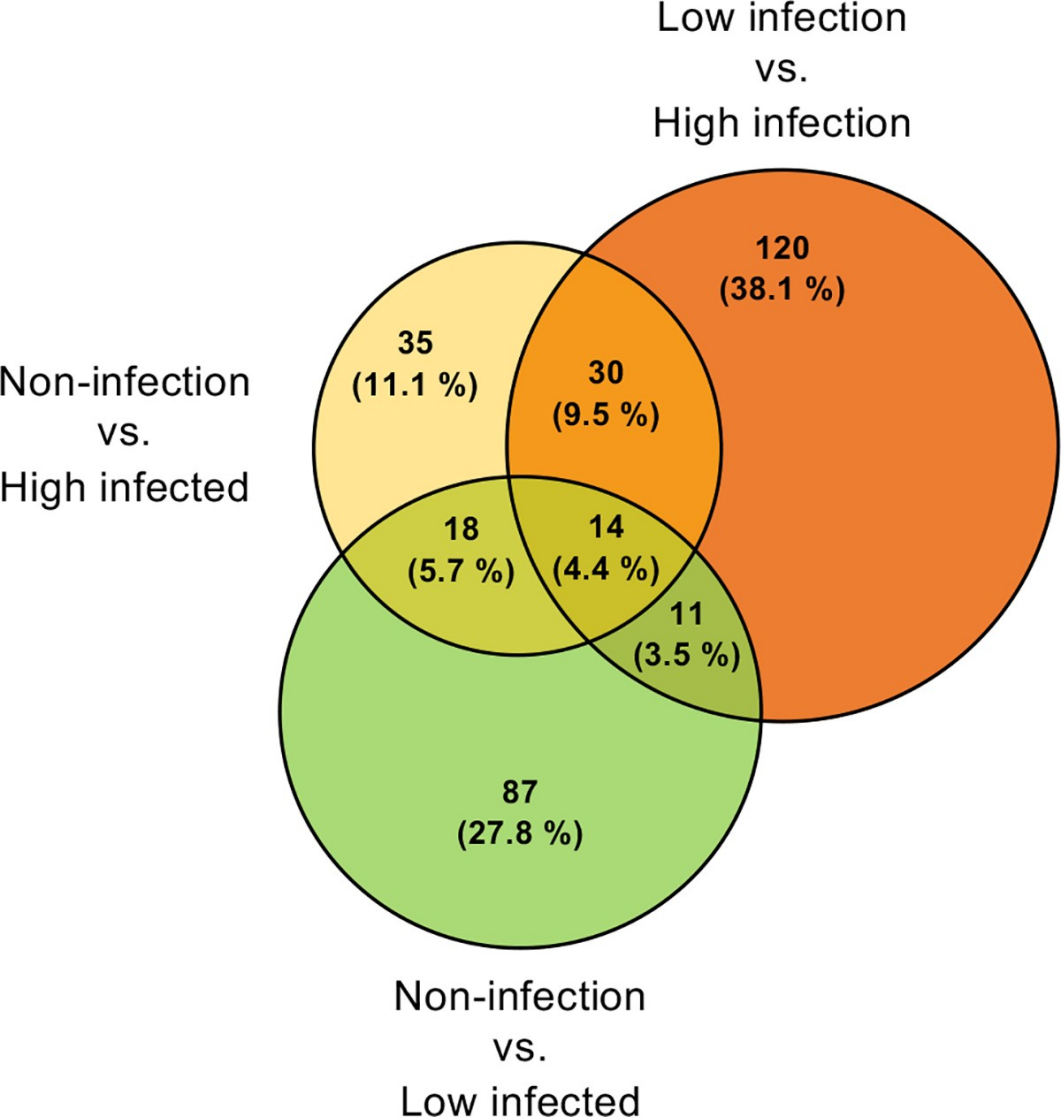
Dengue infection induces differential transcript abundances. Proportional Venn diagram depicting all pairwise comparisons (n = 402) among the antennal transcriptomes of non-infected, low and highly infected females (DENV-1 infection at 14 days post-infection). Overlapping regions represent the subsets of transcripts that are shared among the different conditions. Significant enhancement was determined as a fold change of greater than 1.5, and an FDR-corrected p-value < 0.05 in pairwise comparisons.

Among all the transcripts that were differentially regulated between non-infected and females with a low level of infection (130), a total of 43 transcripts demonstrated higher abundance, while 87 were less abundant in the antennae of non-infected when compared to infected females (Fig 3). Only 97 transcripts were differentially regulated between non-infected and females with a high level of infection, of which 36 exhibited higher abundance, while 61 were less abundant in non-infected mosquitoes (Fig 3). A larger number of transcripts (175) were found to be differentially abundant between females with high and low levels of infection, with 92 transcripts being more abundant and 83 less abundant in highly infected individuals (Fig 3). In total, 297 genes were found to be differentially abundant among the three infection conditions (S1 Data).

**Fig 4:**
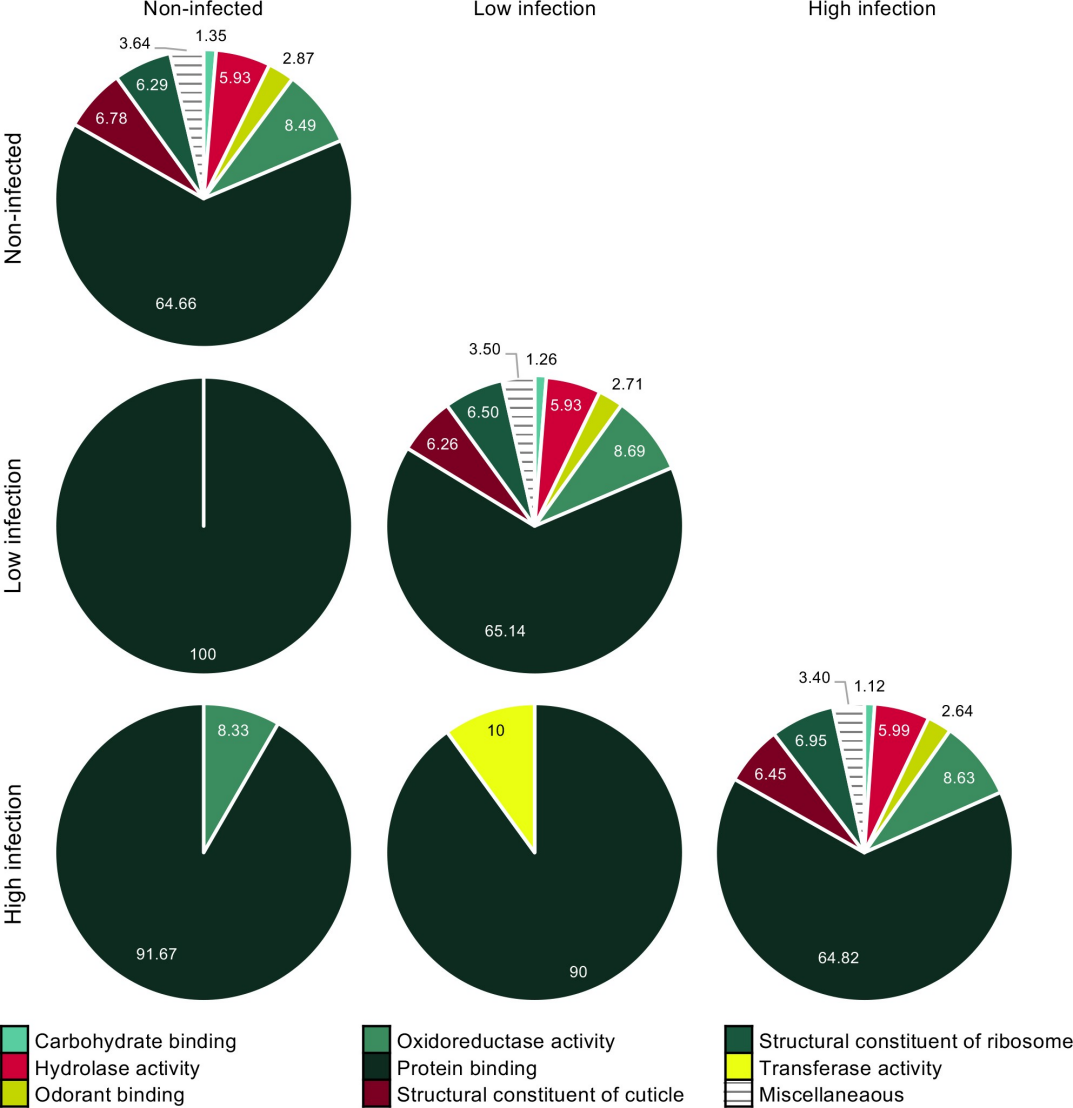
Dengue infection induces differential transcript abundances described by a level three gene ontology analysis of molecular function in the antennal tissue. **A**) The proportion of reliably detected transcripts (n = 16,550) in the antennal transcriptomes of non-infected, low and highly infected females (DENV-1 infection at 14 days post-infection) are presented. **B**) The proportion of differentially abundant transcripts (n = 402) among pairwise comparisons of the three conditions are presented in a matrix.

A gene ontology (GO) analysis of molecular function (level three) described similar proportions among functional classes of all the transcripts that were reliably identified in the antennae of both non-infected and 14 dpi females (Fig 4 diagonal). Of the 315 differentially abundant transcripts, the only functional class shared among all of the conditions was “protein binding” (GO:0005515) (Fig 4), with 41% and 13% of the transcripts involved in protein dimerization activity (GO:0046983), and in calmodulin binding (GO:0005516), respectively, with most transcripts being more abundant in 14 dpi mosquitoes compared to non-infected insects (S2 Data). In addition, the functional classes “oxidoreductase activity” (GO:0016491) and “transferase activity” (GO:0016740) were differentially abundant between non-infected and highly infected, and between females with low and high levels of infection (Fig 4), respectively, with all transcripts being more abundant in mosquitoes with higher infection load (S2 Data). A transferase of note is glutathione S–transferase (GST), which has a presumed xenobiotic, including odorant degrading enzyme, activity. “Odorant binding” (GO:0005549) was a prominent functional class identified in the antennae of 14 dpi and non-infected mosquitoes, but contained no transcripts that were differentially abundant (Fig 4).

**Fig 5:**
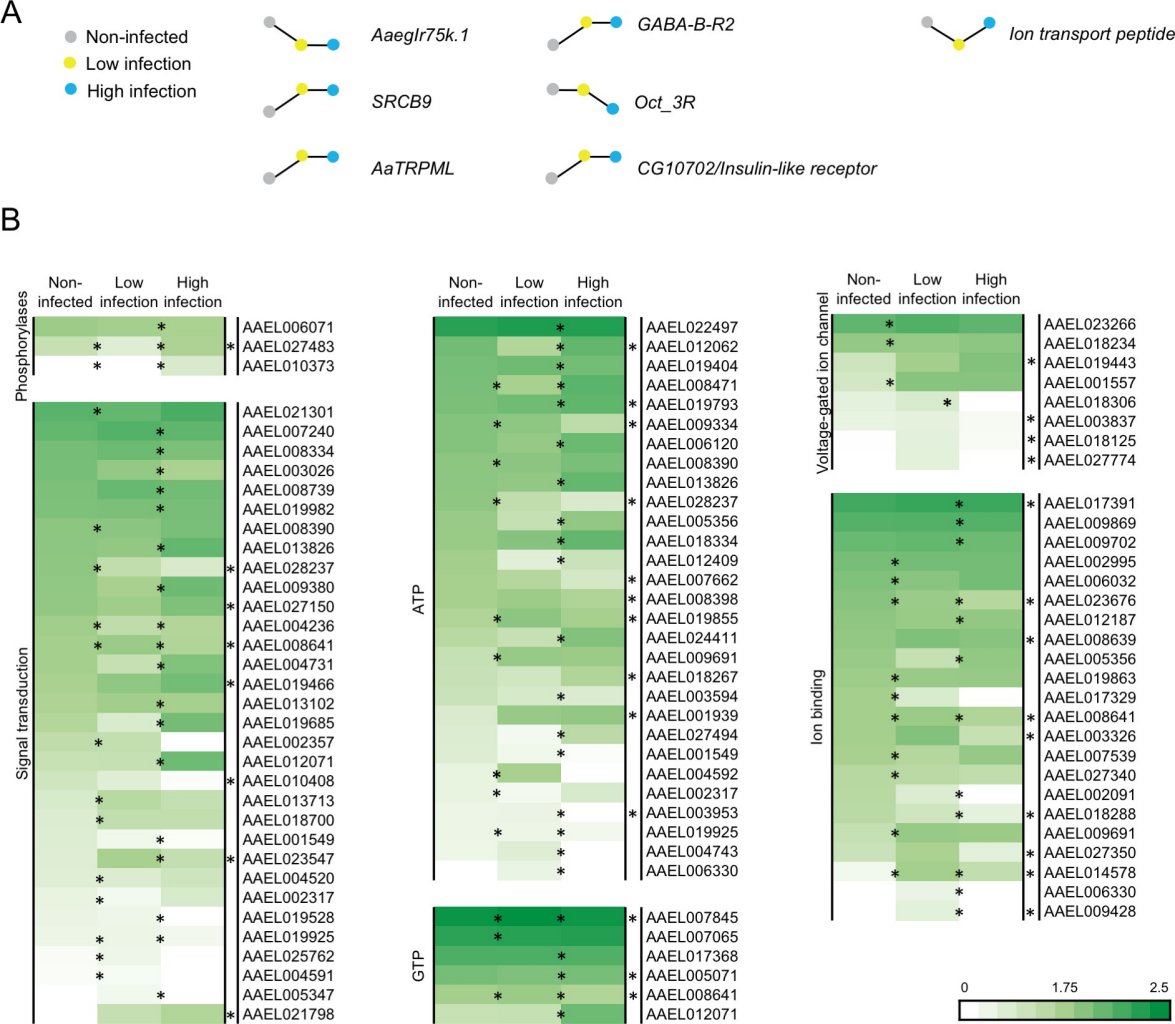
Dengue infection induces differential transcript abundances in key gene families. (A) Differentially abundant chemosensory (left) and neuromodulatory receptor (middle), and neuropeptide (right) genes, represented by stick and ball diagrams, indicating relative levels of abundance at different levels of infection. (B) The heat plots indicate differentially abundant neural signalling genes in the antenna of females with different levels of infection. Significantly different abundance is indicated by asterisks between conditions (fold change > 2; FDR-corrected P < 0.05). An asterisk on the far right of the heat plots indicates comparisons between non-infected and highly infected conditions.

A more in-depth analysis of the genes from families described as chemosensory (S1 and S2 Figs), however, revealed a low number of differentially abundant transcripts, including *AaegIr75k*.*1*, *SRCB9*, and *AaTRPML* (Fig 5). Other genes implicated in the regulation of odor-mediated behavior in insects, including those involved in neurotransmitter, neuropeptide and biogenic amine signaling (S3 Fig), were reliably detected, with only the *CG10702/Insulin-like* neuropeptide receptor, the neurotransmitter receptor *GABA-B-R2*, the ion transport peptide *AAEL019725*, and the biogenic amine receptor *Oct-3R* being differentially abundant across different infection states (Fig 5A).

To further identify genes of interest, a more in depth gene ontology analysis of the differentially abundant transcripts revealed a substantial number of genes involved in neural signaling pathways, including phosphorylase activity (3), signal transduction (32), ATP (29) and GTP (6) activities, voltage-gated ion channel (8) and ion binding (22) (Fig 5B; S3 Data). In all of these functional categories, there was a significantly higher proportion of differentially abundant transcripts in the antennae of 14 dpi females compared to their non-infected counterparts (Fig 5B).

## Discussion

Pathogen infection has previously been shown to alter the behavior of female mosquitoes to enhance the transmission of disease [23,24,26,32]. Female mosquitoes rely heavily on their sense of smell to locate human hosts [13,50], and in this study we demonstrate that 14 dpi mosquitoes are more sensitive to human odor than their 6 dpi counterparts. While 6 dpi females are more active, 14 dpi mosquitoes are more targeted in their search for human odor, emphasizing that the dengue virus is selectively affecting the vector to enhance virus transmission, similar to other pathogens [1,3,22]. Illumina sequencing of antennal transcripts did not identify the chemosensory or neuromodulatory gene families as the major targets of this enhanced sensitivity to human odor, but rather revealed genes involved in neural signaling pathways, as previously demonstrated in whole body transcriptome analysis of *Plasmodium falciparum* infected malaria mosquitoes [51]. The coincident increase in sensitivity to human odors of mosquitoes and their competence to transmit dengue to another human, has dire implications for vectorial capacity.

The four dengue serotypes [52, 53], including one of the most infectious serotypes, DENV-1 [40], have similar extrinsic incubation periods [54], with a high viral titer in the midgut and abdomen by 7–11 dpi [24,31]. In contrast with the previous hypothesis that mosquito behavior post-infection is a response to any challenge to the immune system [9,55,56], locomotion has been shown to be affected in a pathogen-specific manner by several infectious agents. For example, *Wolbachia pipientis-* and DENV-1-infected *Ae*. *aegypti* exhibit increased locomotor activity [18, this study], whereas Zika-infected females display no change in activity within the first week post-infection [57]. The observed increase in locomotion, without changing the sensitivity to human odor, may represent an active manipulation by the virus to increase viral transmission, by increasing the spatial exploration of infected mosquitoes, as demonstrated in other insect systems [58,59]. The demonstrated effect on locomotor activity in this study is likely to be an underestimate, as only ca. 80% of the females tested were infected with DENV-1. While the mechanism for such manipulation is unclear, by 6-dpi the thoracic ganglia, the neuropils regulating locomotion in insects, have been invaded by the virus [60]. Moreover, by this time the midgut tissues have upregulated the transcription of several modulators, including biogenic amines [61], which are known to modulate locomotion in response to infection [62]. Considering the different behavioral phenotypes displayed by infected mosquitoes, pathogens appear to exert different strategies against their hosts to achieve transmission.

Dissemination of the virus into the salivary glands by 14–16 dpi [24,31] is essential for dengue transmission to a human host [63,64]. This coincides with the invasion into the head tissues, including the antennae [31,33], and an increased behavioral and physiological sensitivity to human odor (this study). This progression of infection into the salivary glands, coinciding with an increased attraction to the human host, is reflected in the interaction between *Plasmodium yoelii* parasite and *Anopheles stephensi* malaria mosquitoes [20,27], as well as between *Ae*. *aegypti* and *Plasmodium gallinaceum* parasites [22]. However, this does not appear to be the case for *Plasmodium falciparum* and *An*. *gambiae* [65,66], or for West Nile virus and *Culex pipiens*, in which transmission competent females are less responsive to human odors post-infection [18,67]. While 14 dpi females were significantly more active in response to human odour, the mosquitoes demonstrated a decreased locomotor activity during peak host seeking periods. This suggests that the pathogen may play an active role in increasing the risk of transmission by both increasing sensitivity to human odor and decreasing the risk of vector mortality.

To identify the potential molecular mechanism regulating the observed increase in sensitivity to human odor in 14 dpi females, the transcript abundance in the primary olfactory tissue, the antennae, was assessed in non-infected females and those infected with low and high levels of DENV-1. The high infection level used in this study is in accordance with virus titers detected naturally in mosquitoes [40]. A gene ontology analysis of molecular function associated with the antennal transcripts identified oxidoreductase and transferase activity, but not odorant binding, as affected 14 dpi with DENV-1, with higher transcript abundance in the antennae of infected females with natural levels of infection. A further analysis of the specific transcripts identified as differentially abundant within the oxidoreductase and transferase activity classes were found to be primarily involved in neural signaling. A comparison of the differential abundance patterns of these neural signaling genes with those from a previous study [9] demonstrated that all but one (AAEL003837) were differentially regulated at 14 dpi in the antennae but not in the midgut, salivary gland or whole-body transcriptomes. While dengue infection generally results in a decrease in transcript abundance across various tissues [9,68,69], the opposite was found in the antennae. Such tissue-specific transcript change in response to arboviral infection progresses differentially over time, reflecting the viral temporal tropism [9,68,70]. This selective increase in the transcript abundance of genes involved in neural signaling pathways provides a mechanism that has the potential to generate the increased physiological and behavioral sensitivity to human odor post-infection.

Other genes identified to be differentially regulated in the antennae post-infection include a neuromodulator, ion transport peptide, and three neuromodulatory receptors, insulin-like receptor, GABA-B receptor and octopamine receptor, none of which are differentially regulated in other tissues [9,55,68,70]. Both GABA and octopamine signaling are involved in regulating olfactory sensitivity in insects [71,72], and the manipulation of such pre-existing signaling pathways is one way in which pathogens and parasites regulate the behaviors of their hosts [73,74]. While the role of the ion transport peptide in response to infection is currently unknown, insulin signaling in insects has received considerable attention due to its role in sensing and responding to metabolic state through many channels, such as regulating olfactory sensitivity [75,76]. Moreover, insulin-like peptides show a systemic increase following viral infection and are involved in reducing titers of West Nile, Zika and dengue viruses in mosquitoes [77]. This is intriguing in the light of the increased abundance of the insulin-like receptor in the antennae of females 14 dpi. However, the significance of the differential expression of these genes and their function in antennae of 14 dpi mosquitoes, particularly with respect to regulating the sensitivity of the peripheral olfactory system, remains unclear.

Contrary to what has been described previously [33,78], only three members of the chemosensory gene families were differentially regulated in response to a change in infection status. While previous studies have demonstrated a connection between behavioral sensitivity and an increased transcript abundance of select ORs and OBPs [33,34], this study identified *IR75k*.*1* as the only chemosensory gene to change (decrease) in response to DENV-1 infection. The orthologue, AgamIR75k, in *Anopheles gambiae* has been shown to respond to short chain carboxylic acids [79,80], which regulate host seeking in mosquitoes [81,82]. While it is known that the malaria parasite, *Plasmodium falciparum*, alters the odor profile of infected humans [83,84], including changing the emission of select carboxylic acids [85], similar studies are lacking for dengue infection. The potential modulation of mosquito responsiveness to odor emissions from human hosts by DENV-1 will be of interest for further investigation.

DENV-1 differentially affects the physiology and behavior of *Ae*. *aegypti* reflecting the viral temporal tropism and the bimodal regulation exerted by this pathogen. While 4–6 dpi mosquitoes are more active during both late photophase and into early scotophase, these females do not display a change in attraction to human odor. The mechanism underlying this change in behavior is yet unknown, but is likely to increase the mobility and local spread of infected mosquitoes. In contrast, 14 dpi females are less active during their natural host seeking period, but at the same time, display an increased physiological and behavioral sensitivity to human odor, linked to an antenna-specific increase in the abundance of predominantly neural signaling genes. This modulation in sensitivity coincides with the progression of DENV-1 into the salivary glands [31], and the associated change in blood feeding pattern [24,32]. The increased propensity to host seek, together with extended feeding periods from multiple hosts intensifies the interaction between infective mosquitoes and humans, inadvertently enhancing the vectorial capacity, and thus increasing the risk of DENV-1 transmission.

## Supporting information

S1 Fig Transcript abundance of odorant binding and degrading proteins. Transcript abundance of odorant binding protein (OBP; A), chemosensory protein (CSP; B) and odorant degrading enzyme (ODE; C) genes in the antenna of 19 days post-emergence *Aedes aegypti* females, either non-infected or with differing levels of DENV-1 infection after 14 days post-infection.
(TIFF)

S2 Fig Transcript abundance of antennal membrane associated proteins. Transcript abundance of odorant receptor (OR; A), ionotropic receptor (IR; B), gustatory receptor (GR; C) sensory neuron membrane protein (SNMP; D), pickpocket (PPK; E), and transient receptor protein (TRP; F) genes in the antenna of 19 days post-emergence *Aedes aegypti* females, either non-infected or with differing levels of DENV-1 infection after 14 days post-infection. Significantly different abundance is indicated by asterisks between conditions (fold change > 2; FDR-corrected P < 0.05).
(TIFF)

S3 Fig Transcript abundance of neuromodulatory genes. Transcript abundance of neuropeptide (A), neuropeptide receptor (B), biogenic amine-related enzymes (C), biogenic amine receptor (D), transporter (E) and neurotransmitter receptor (F) genes in the antenna of 19 days post-emergence *Aedes aegypti* females, either non-infected or with differing levels of DENV-1 infection after 14 days post-infection. Significantly different abundance is indicated by asterisks between conditions (fold change > 2; FDR-corrected P < 0.05). An asterisk on the far right of the heat plots indicates comparisons between non-infected and highly infected conditions.
(TIFF)

S1 Table Lists the total number of reads, mapped reads (%), transcripts and reliably detected transcripts (threshold > 1 TPM) for the transcriptomes of each of the biological replicates of non-infected, and females identified as having low (Low) and high (High) levels of DENV-1 infection at 14 days post-infection (dpi).
(XLSX)

S1 Data Lists the permanent identifier in vectorbase, gene name and name of the *Drosophila* homologue. (XLSX)

S2 Data Lists the permanent identifiers, transcript abundance (TPM), directional comparison of differential abundance, fold change and FDR-corrected p-value for transcripts identified among the DENV-1 infection conditions. (XLSX)

S3 Data Lists the functional category, gene names, permanent identifiers and putative functions of the differentially abundant neural signalling genes identified among all of the DENV-1 infection conditions. (XLSX)

S4 Data The viral load of DENV-1. The viral load of DENV-1 for each infected mosquito, included in the analysis, as determined by quantitative polymerase chain reaction (qPCR).
(XLSX)
