# Supplementary figures and images for: Dengue infection modulates locomotion and host seeking in Aedes aegypti
Source: PLoS Negl Trop Dis. 2020 Sep 10;14(9):e0008531. doi: 10.1371/journal.pntd.0008531 (PMC7482838; doi:10.1371/journal.pntd.0008531)

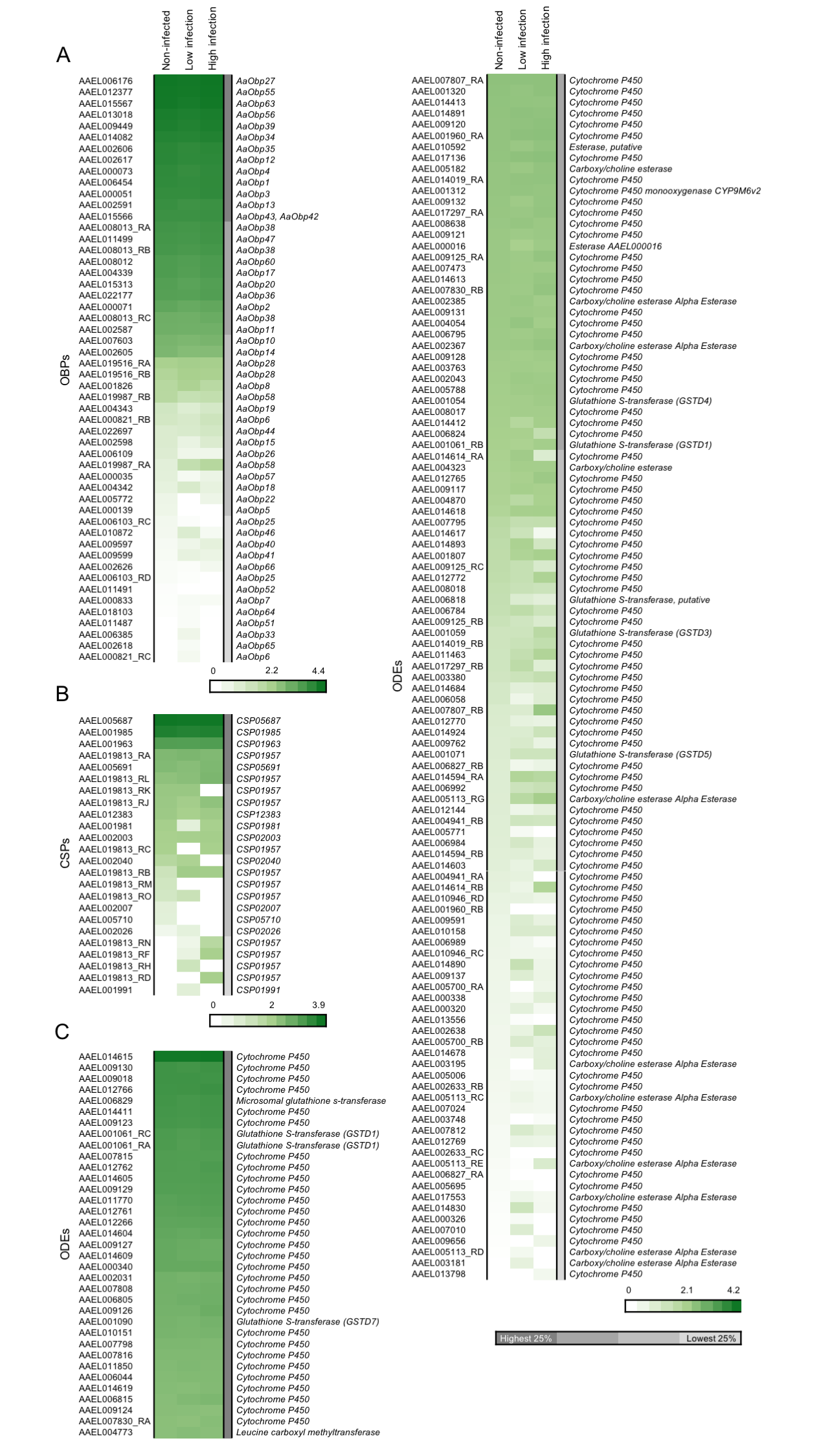

Supplement: S1 Fig — Transcript abundance of odorant binding protein (OBP; A), chemosensory protein (CSP; B) and odorant degrading enzyme (ODE; C) genes in the antenna of 19 days post-emergence Aedes aegypti females, either non-infected or with differing levels of DENV-1 infection after 14 days post-infection. (TIFF) [file pntd.0008531.s001.tiff]

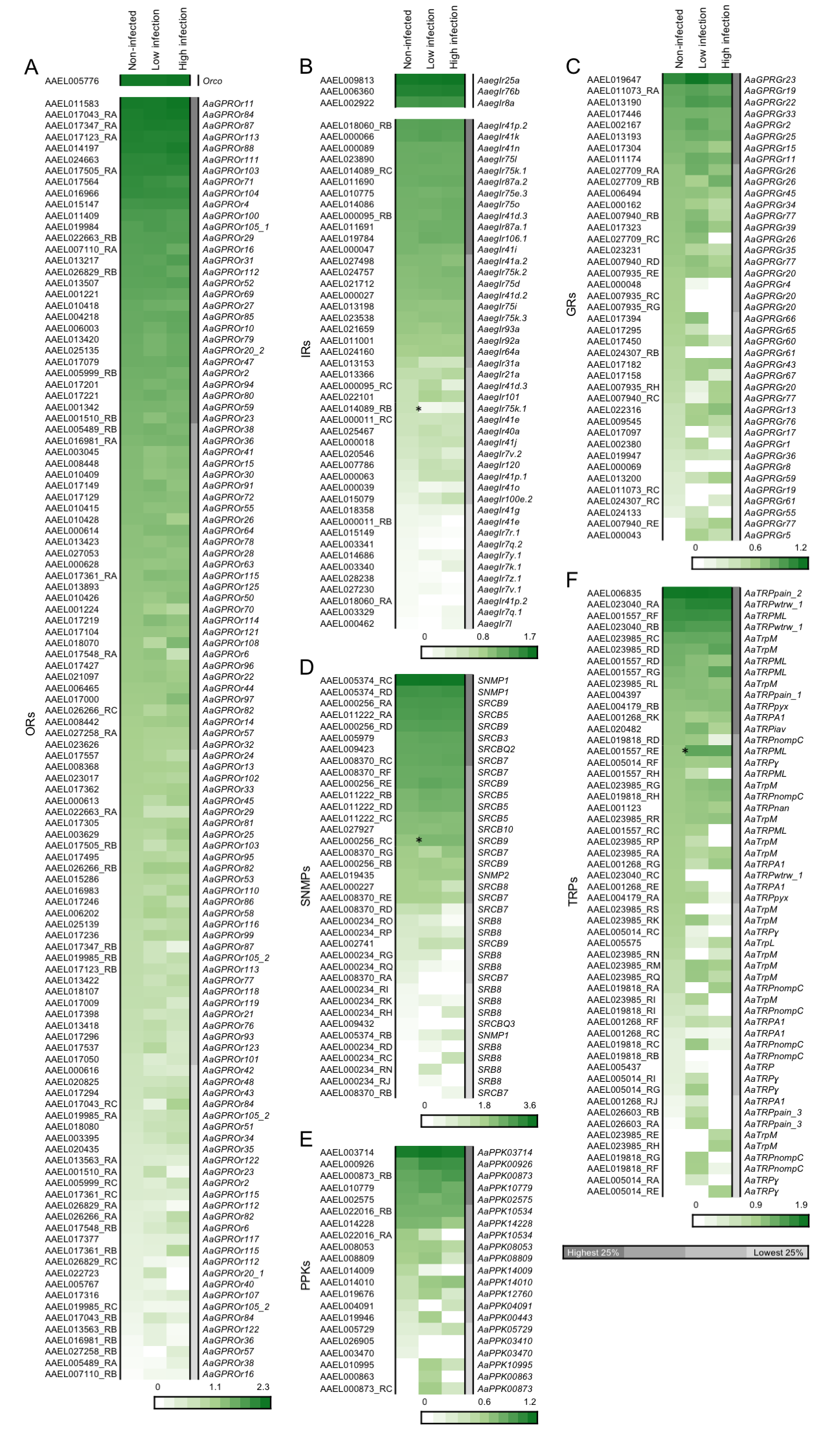

Supplement: S2 Fig — Transcript abundance of odorant receptor (OR; A), ionotropic receptor (IR; B), gustatory receptor (GR; C) sensory neuron membrane protein (SNMP; D), pickpocket (PPK; E), and transient receptor protein (TRP; F) genes in the antenna of 19 days post-emergence Aedes aegypti females, either non-infected or with differing levels of DENV-1 infection after 14 days post-infection. Significantly different abundance is indicated by asterisks between conditions (fold change > 2; FDR-corrected P < 0.05). (TIFF) [file pntd.0008531.s002.tiff]

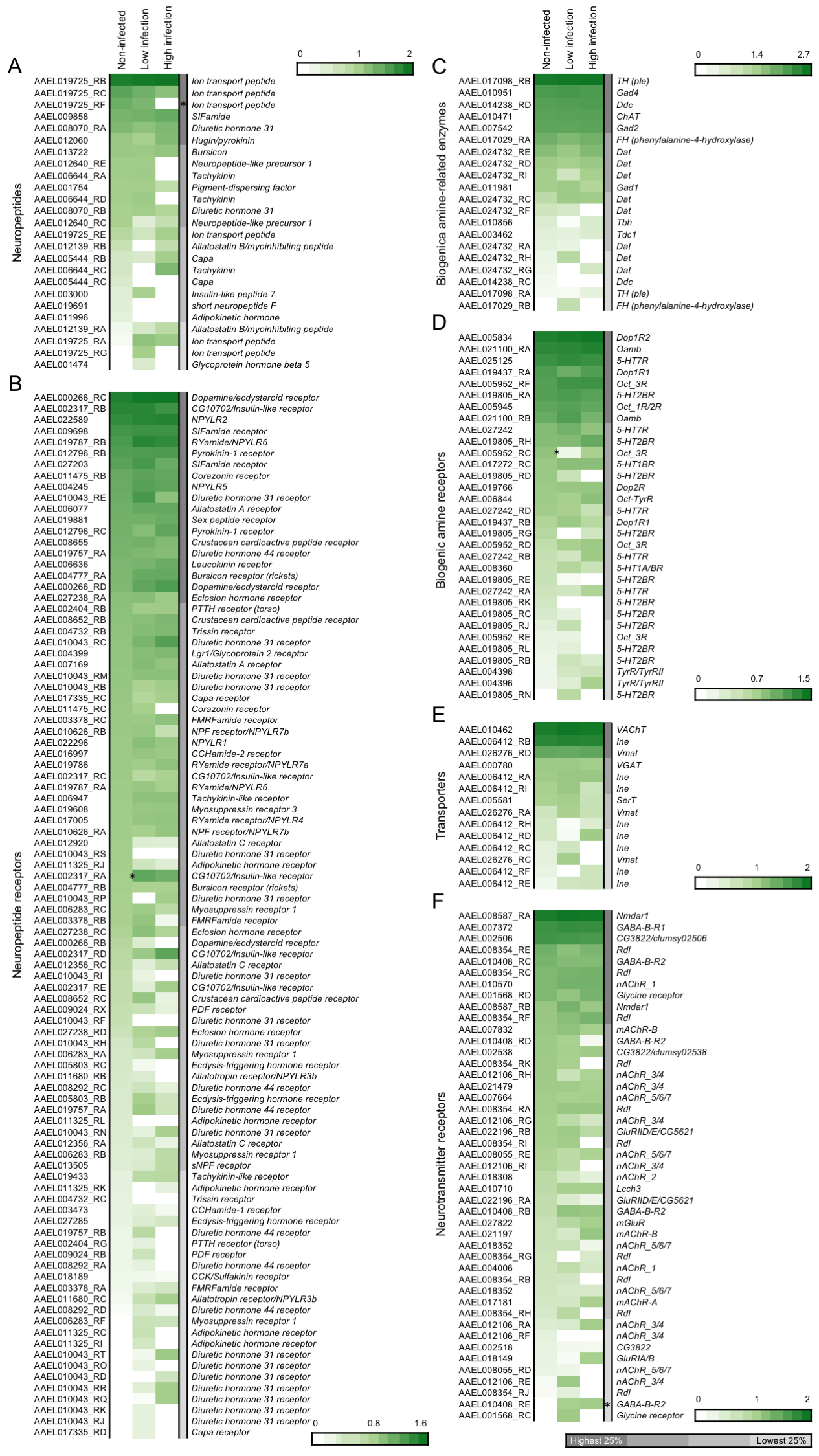

Supplement: S3 Fig — Transcript abundance of neuropeptide (A), neuropeptide receptor (B), biogenic amine-related enzymes (C), biogenic amine receptor (D), transporter (E) and neurotransmitter receptor (F) genes in the antenna of 19 days post-emergence Aedes aegypti females, either non-infected or with differing levels of DENV-1 infection after 14 days post-infection. Significantly different abundance is indicated by asterisks between conditions (fold change > 2; FDR-corrected P < 0.05). An asterisk on the far right of the heat plots indicates comparisons between non-infected and highly infected conditions. (TIFF) [file pntd.0008531.s003.tiff]
